# Data Independent Acquisition Mass Spectrometry Can Identify Circulating Proteins That Predict Future Weight Loss with a Diet and Exercise Programme

**DOI:** 10.3390/jcm8020141

**Published:** 2019-01-25

**Authors:** Nagaraj Malipatil, Helene A. Fachim, Kirk Siddals, Bethany Geary, Gwen Wark, Nick Porter, Simon Anderson, Rachelle Donn, Michelle Harvie, Anthony D. Whetton, Martin J. Gibson, Adrian Heald

**Affiliations:** 1 The School of Medical Sciences and Manchester Academic Health Sciences Centre, University of Manchester, Manchester M13 9PL, UK; nagaraj.malipatil@nhs.net (N.M.); kirk.siddals@manchester.ac.uk (K.S.); bethany.geary@manchester.ac.uk (B.G.); simon.anderson@manchester.ac.uk (S.A.); rachelle.donn@manchester.ac.uk (R.D.); tony.whetton@manchester.ac.uk (A.D.W.); martin.gibson@manchester.ac.uk (M.J.G.); 2 Department of Diabetes and Endocrinology, Salford Royal Hospital, Salford M6 8HD, UK; 3 Stoller Biomarker Discovery Centre, University of Manchester, Manchester M13 9PL, UK; 4 SAS Peptide Hormone Section, Part of Berkshire and Surrey Pathology Services, Royal Surrey County Hospital, Guildford GU2 7XX, UK; gwen.wark@nhs.net (G.W.); nick.porter@nhs.net (N.P.); 5 The George Alleyne Chronic Disease Research Centre, Caribbean Institute for Health Research (CAIHR), The University of the West Indies, Bridgetown BB11115, Barbados; 6 The Prevent Breast Cancer Research Unit, The Nightingale Centre, Manchester University NHS Foundation Trust, Manchester M23 9LT, UK; michelle.harvie@manchester.ac.uk; 7 Manchester Breast Centre, Manchester Cancer Research Centre, University of Manchester, 555 Wilmslow Rd, Manchester M20 4GJ, UK; 8 NIHR Manchester Biomedical Research Centre, University of Manchester, Manchester M13 9WU, UK

**Keywords:** IGR, lifestyle change, proteomics, SWATH MS

## Abstract

We investigated biological determinants that would associate with the response to a diet and weight loss programme in impaired glucose regulation (IGR) people using sequential window acquisition of all theoretical fragment ion spectra (SWATH) mass spectrometry (MS), a data acquisition method which complement traditional mass spectrometry-based proteomics techniques. Ten women and 10 men with IGR underwent anthropometric measurements and fasting blood tests. SWATH MS was carried out with subsequent immunoassay of specific peptide levels. After a six-month intervention, 40% of participants lost 3% or more in weight, 45% of patients remained within 3% of their starting weight and 15% increased their weight by 3% or more. Hemoglobin A1c (HbA1C) level was reduced with weight loss with improvements in insulin sensitivity. SWATH MS on pre-intervention samples and subsequent principal component analysis identified a cluster of proteins associated with future weight loss, including insulin-like growth factor-II (IGF-II) and Vitamin D binding protein. Individuals who lost 3% in weight had significantly higher baseline IGF-II levels than those who did not lose weight. SWATH MS successfully discriminated between individuals who were more likely to lose weight and potentially improve their sensitivity to insulin. A higher IGF-II baseline was predictive of success with weight reduction, suggesting that biological determinants are important in response to weight loss and exercise regimes. This may permit better targeting of interventions to prevent diabetes in the future.

## 1. Introduction

There remains uncertainty as to what are the determinants of progression from impaired glucose regulation (IGR) or pre-diabetes to type 2 diabetes (T2MD) particularly in relation to what may assist in the prediction of response to lifestyle interventions.

There is strong evidence to suggest that without any lifestyle or medical intervention, in particular weight loss and physical activity, more than 50% of people with IGR will develop T2DM accompanied by an increased risk of cardiovascular disease and cardiovascular death over a period of 10 years [1]. In many areas of the UK, people with impaired glucose regulation (IGR) are being identified and they are now being offered a lifestyle change through the National Diabetes Prevention Programme (NDPP) [2].

In Salford UK, we have a lifestyle change programme called Care Call (http://clahrc-gm.nihr.ac.uk/resources/igt-care-call/) [3], a telephone based modular intervention programme, which has been proven to benefit people with impaired glucose regulation. Results in terms of weight reduction and fall in hemoglobin A1c test (HbA1c), over time are variable [4,5,6].

Proteins are the principal effectors of cellular mechanisms and are key players in the maintenance of cellular homeostasis. Consequently, the disruption of their physiological functions is usually associated with several disease phenotypes. In this sense, the study of these proteins and their functions has been extremely important in order to decipher the cellular mechanisms and organization of particular phenotypes [7,8].

Sequential window acquisition of all theoretical fragment ion spectra (SWATH) Mass Spectometry (MS) is a data independent acquisition (DIA) method, which aims to complement traditional mass spectrometry-based proteomics techniques such as shotgun and selection reaction monitoring (SRM) methods [9]. In essence, it allows a complete and permanent recording of all fragment ions of the detectable peptide precursors present in a biological sample [10].

We herein set out to determine using this biological ‘fingerprinting’ technique for the first time in this area, if we can identify individuals who are more likely to lose weight with a validated lifestyle change intervention.

## 2. Methods

Twenty individuals (10 female, 10 male) with IGR living in Salford (UK) were consecutively recruited. All participated in the Care Call Programme. This is a modular intervention programme utilising motivational support techniques, lifestyle education, one to one and peer discussion and encouragement of progress with goals and signposting/referral to relevant services, with tailoring of content to individual needs.

Salford Royal’s Diabetes Care Call is a telephone-based service available for people with diabetes that has been further developed to support people diagnosed with Impaired Glucose Tolerance (IGT) and are most at risk from developing type 2 diabetes. Care Call development provides people with a six-month programme of education and motivational support which is delivered by a team of health advisors using the telephone.

The Care Call programme is a structured pathway that provides to the patients eight appointments by telephone with personal and dietary advices; information about diet, lifestyle and activity, including tips that can help the patients to reduce the risk to develop T2DM.

In addition, an “Eatwell guide” helps the patients to understand which different types of food they should aim to eat each day and what portion sizes should be. Additionally, the health advisors help the patients to become more active. When patients are suitable, the advisors can refer them to any Fit City Centre located in Salford and offer a free eight weeks pass.

Participants had weight and height recorded and underwent anthropometric measurements including waist measurement and waist-hip ratio before the start of the six month intervention, and after its completion. Fasting blood tests were carried out at these time points. At each visit, blood was taken into lithium heparin tubes and into serum tubes, spun down, aliquoted and stored at −80 °C for future analysis.

SWATH MS was carried out using SCIEX (Framingham, Boston, MA, USA) 6600 instrumentation. Plasma was centrifuged at 4 °C (15700× g for 5 min) to remove particulates. Major plasma proteins were removed by immunodepletion using Pierce Top 12 abundant protein depletion spin columns. Plasma samples were concentrated using a MiVac vacuum centrifuge Genevac™ (Thermo Fisher Scientific, Loughborough, UK) and the protein concentration measured using the Pierce 660 nm Protein Assay (Thermo Fisher Scientific, Warminster, PA, USA). Depleted plasma (50 μg) was reduced, alkylated and digested overnight at 37 °C with sequencing gradetrypsin (Promega, Fitchburg, WI, USA). After drying and reconstitution, 10 μg of the sample was loaded onto the microflow LC-MS system and analysed, as previously described [11].

HbA1c was measured on the Menarini 9210 Premier automated analyser (boranate affinity and high performance liquid chromatography), (Menarini, Wokingham Berkshire, UK). Serum fasting insulin was assayed using the Mercodia ELISA (Uppsala, Sweden). Fasting blood glucose was determined.

Patient serum samples and controls containing protein bound IGF-II were extracted with acid-ethanol to precipitate the binding proteins. TRIS was incubated with the supernatant to yield a second precipitate and neutralise the acid used in the previous step. The neutralised sample was then diluted to allow measurements to be made within the assay range.

The IGF1 and IGFBP3 were analysed on the IDS iSYS analyser using IDS iSYS reagents (Immunodiagnostic Systems Holdings PLC, Tyne & Wear, UK).

The IGF-II in the diluted extract competes with 125I-IGF-II for the limited binding sites on a specific mouse monoclonal antiserum (Millipore, Burlington, MA, USA). Incubation was at 2–8 °C overnight. Antibody fractions were precipitated by the addition of anti-mouse gamma-globulin (Millipore, Burlington, MA, USA) in a polyethylene glycol (PEG) phosphate buffered solution.

The homeostasis model assessment (HOMA-S), a measure of insulin sensitivity, was estimated using the fasting insulin and fasting glucose measurements [12] using the calculator available at https://www.dtu.ox.ac.uk/homacalculator/.

All subjects gave their informed consent for inclusion before they participated in the study. The study was conducted in accordance with the Declaration of Helsinki, and the protocol was approved by the Ethics Committee of Health Research Authority NRES Committee North West-Greater Manchester West (IRAS project ID: 128954), obtained on 08 October 2014.

### Statistics Analysis

All analyses were carried out using Statistical Package for Social Sciences (SPSS version 20.0, Armonk, NY, USA). Anthropometric measurements were submitted to descriptive statistical analysis. Differences between before and after intervention were examined by paired T test, differences among the categories of weight loss or weight gain (participants were divided into three categories: (i). those who lost 3% or more, (ii). those who remained within 3% of their starting weight, (iii). those who increased their weight by 3% or more) were analysed by one way ANOVA.

A value of *p* = 0.05 was used as the threshold for statistical significance.

The IGF-I/IGFBP-3 molar ratio was calculated based on a molecular mass of 7.6 kDa for IGF-I and 29 kDa for IGFBP-3, respectively.

## 3. Results

The anthropometric measurements taken of the patients before and after the intervention are shown in Table 1. The descriptive analysis shows the mean age of the participants before the intervention (61.5 ± 4.83, male; 60.2 ± 2.73, female), initial weight (108.2 ± 9.18, male; 94.8 ± 5.16, female), height (175.8 ± 2.85, male; 161.9 ± 2.46, female), hip-waist ratio (1.00 ± 0.02, male; 0.90 ± 0.01, female) and body mass index (BMI) (35.0 ± 2.88, male; 36.2 ± 1.94, female).

By the end of the sixth month of lifestyle intervention, specifically in percentage terms, individuals in general lost on average 1% of their total body weight (t = 2.43; *p* = 0.025). The weight loss and BMI changes were statistically significant in female (t = 2.60, *p* = 0.02; t = 2.55, *p* = 0.03) (Table 1).

The clinical variables (high-density lipoprotein (HDL), low-density lipoprotein (LDL), triglycerides and insulin) levels before and after six months of intervention are shown in the Table 2. Patients showed no differences in any of those variables after intervention (Table 2).

Dividing by categories, 40% of patients lost 3% or more in weight, 45% of patients remained within 3 % of their starting weight and 15% increased their weight by 3% or more.

**Figure 1:**
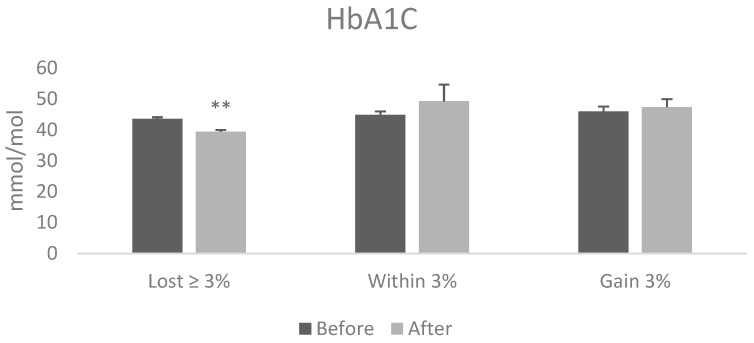
Hemoglobin A1c (HbA1c) levels before and after the intervention among patients who lost ≥3% in body weight, patients who remained within 3% of their starting weight and those who increased their weight by 3% or more. The values before and after within the groups were analysed by Paired T test and are shown as mean ± SE. ** *p* < 0.01.

Among those who lost ≥3%, HbA1C levels significantly reduced (from 43.6 to 39.5) after intervention (t = 4.101, *p* = 0.006) (Figure 1). Consistent with this result, although not achieving significance, were improvements in insulin sensitivity in this group (HOMA-S from 61.5% ± 13.4% to 50.2% ± 12.2%); (HOMA-B from 135% ± 24.1% to 117% ± 18.1%) following the intervention. Those patients who remained within 3% and those who gained weight also did not present significant changes (HOMA-S from 76% to 103% ± 27%; HOMA-B from 133% to 110% ± 16%).

**Figure 2:**
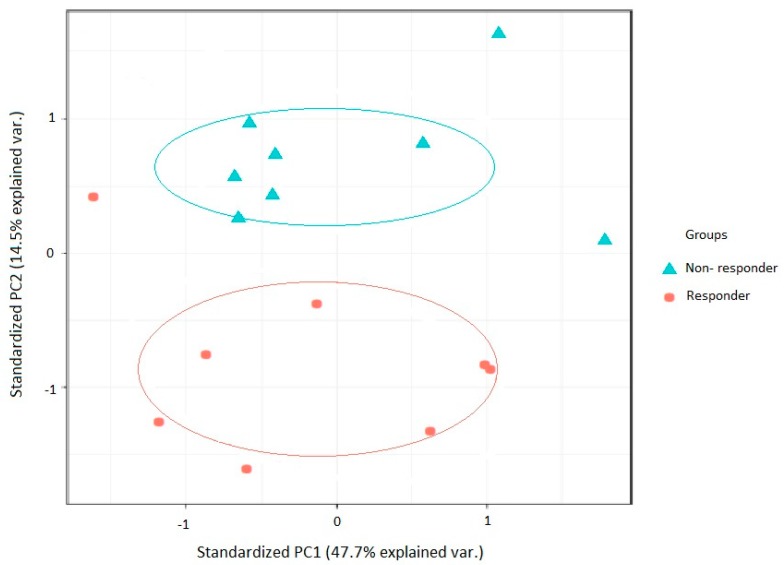
Principal component analysis of proteins that were potentially predictive of weight reduction of 3% or more in 20 people with impaired glucose regulation (IGR). Principal component analysis of data derived on the initial pilot samples. These data demonstrate the discriminatory power of SWATH MS in separating the two groups (future weight loss vs no significant weight loss).

Principal component analysis (PCA) of data derived on the data derived from samples in relation to the outcome of weight loss. These data demonstrate the discriminatory power of SWATH MS in relation to identifying the major proteins responsible for the separation of the two groups of 3% or more subsequent weight loss vs no significant weight loss (Figure 2).

The proteins that we analysed in the PCA discriminating future weight loss vs no significant weight loss included IGF-II and Vitamin D binding protein. The complete list of the proteins is given in Supplementary Table 1.

**Figure 3:**
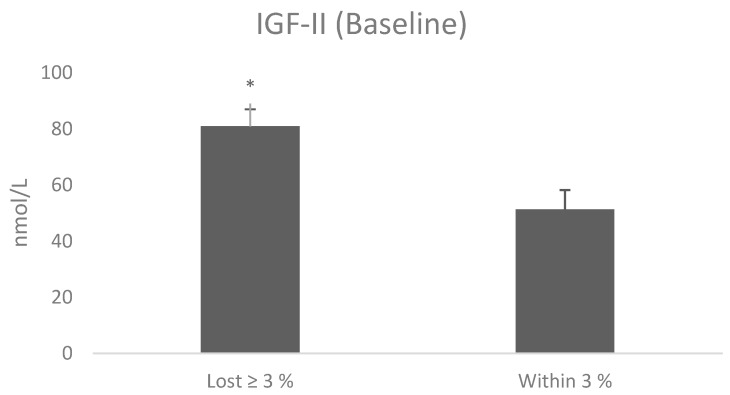
Insulin-like growth factor II (IGF-II) baseline levels between patients who lost ≥3% in body weight and those patients who remained within 3% of their starting weight. The values were analysed by T test and are shown as mean ± SE. * *p* < 0.05.

**Figure 4:**
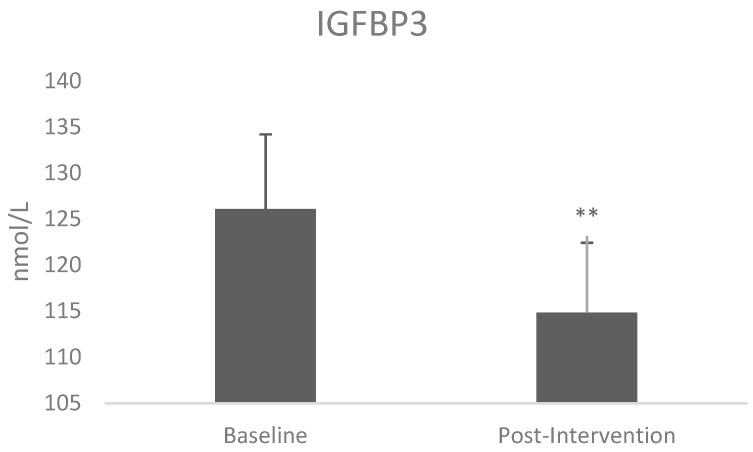
Insulin-like growth factor binding protein 3 (IGFBP3) levels changes following intervention. The values were analysed by paired T test and are shown as mean ± SE. ** *p* < 0.01.

Immunoassay of IGF-II showed no differences pre- and post-intervention, however, individuals who lost ≥3% had significantly higher baseline IGF-II levels (81 nmol/L; *p* = 0.024) than those that remained within 3% (51.35 nmol/L) (Figure 3). There were no differences in IGF-II levels pre- and post-intervention. However there was a reduction of circulating IGFBP-3 following intervention (t = 3.46, *p* = 0.003) (Figure 4) and an increase of IGF-I:IGFBP3 ratio (t = −2.3, *p* = 0.031) (Table 3).

## 4. Discussion

Using the technique of SWATH-MS to generate digitised information we have been able to discriminate individuals who would subsequently lose weight of 3% or more from those who remained weight stable or who gained weight. While the findings are preliminary, they point to the potential utility of this technique in providing a biological ‘fingerprint’ that may guide clinicians and patients with IGR in relation to predicting who is most likely to respond to a lifestyle intervention. A higher IGF-II level was related to a great chance of weight reduction. This is particularly important given that the lifestyle change programme was advisory, not proscriptive.

The panel of proteins identified as discriminating between the groups, included peptides known to be involved in the modulation of weight change/insulin sensitivity over time in individuals with and without T2DM, such as IGF-II [13,14], RBP4 [15,16,17], Fetuin-A [18,19], ZA2G-Zinc-α2-glycoprotein [20], Visfatin [21,22,23] and FAS-Fatty acid synthase [24,25,26]. It also included Vitamin D binding protein whose concentration has been shown to be inversely associated with insulin resistance [27].

The variation in insulin sensitivity change with the Care Call intervention, even for individuals who lost weight is striking. A previous study by Schenk et al, indicated an association between increase in HOMA-S with a weight reduction intervention in IGR individuals and a lower rate of progression to T2DM [28], with reduced fatty acid mobilization and uptake appearing to be a primary mediator of improved insulin sensitivity after weight loss.

SWATH MS has not previously been used to look at IGR individuals in relation to lifestyle intervention, but in other areas of medicine it has been shown to be of prognostic value in relation to disease trajectory, for example in haematological malignancies, lung cancer, colorectal cancer and Parkinson’s Disease [29,30]. Digitised proteomic maps offer great potential as they are applicable to large cohorts and give opportunity for generations of algorithms with discriminatory power for diagnosis, prognosis and theranostics. Our initial data here suggests that this may be the case with respect to dietary intervention and disease.

The lifestyle intervention resulted in decreased IGFBP3 circulating levels. It was demonstrated before that IGFBP-3 can inhibit adipogenic differentiation through direct interaction with PPAR-gamma [31]. Furthermore, studies in animal models show that IGFBP-3 knockout increases adiposity [32] and that IGFBP-3 also affects insulin secretion directly by IGF-independent as well as IGF-dependent mechanisms [33]. IGFBP-3 can also increase circulating glucose levels via inhibition of glucose uptake in adipocytes [34]. These findings reveal an important role for IGFBP-3 in the pathogenesis of obesity and insulin resistance. We also found that the intervention resulted in increased IGF-I:IGFBP3 molar ratio. The molar ratio between total IGF-I and IGFBP-3 (IGF-I/IGFBP-3) has been suggested to indirectly reflect free as well as with bioactive IGF-I [35,36]. Changes in concentrations of IGFBP-3 and the IGF-I:IGFBP-3 ratio deserve attention as they present potential clinical implications suggested by epidemiological data showing significant associations between IGFBP-3 or the IGF-I:IGFBP-3 ratio and various diseases such as cancer, coronary events, hepatic steatosis, and lung function to mortality [37].

Our results demonstrated a gender difference in weight change after the intervention, with women being more likely to lose weight than men. It has been shown before that the difference in the effectiveness of current weight loss programmes is variable between genders [38]. Even though this review brings evidence that men normally lose more weight than women, women also have a significant amount of weight reduction, indicating that men and women should adopt different weight loss strategies.

The Care Call intervention resulted in moderate success with 40% of the participants reducing 3% or more in weight. Metanalysis studies have shown that the effectiveness of interventions for weight loss are variable due to mainly low engagement of the participants rather than the type of intervention [39]. Even though the non-face-to-face programs try to reduce the extrinsic barriers to participation (as lack of time, costs), the lack of motivation may be a key barrier to Diabetes prevention programs engagement [40].

A strength of this study is that we have followed up all 20 individuals who were originally recruited to the study with data collected at baseline and at the six month follow-up on completion of the Care Call intervention, and their consent in donating blood sample and fat tissue. Weaknesses are that the separation between the groups in the PCA was not complete, most probably due to the small sample size, even though it provided significant results in relation to both adipose and blood sample analysis, and the limited degree of weight loss within the study population. Nonetheless the data are encouraging for wider and deeper studies.

## 5. Summary

Using a panel of peptides identified with SWATH MS we have gone some way to be able to characterise the individuals who are more likely to lose weight and potentially improve their sensitivity to insulin when participating in a lifestyle change programme for impaired glucose regulation (IGR). We show also that a higher IGF-II is predictive of weight loss.

Validation of the peptides identified in additional IGR or early T2DM patients undergoing weight loss regimes will lead to a greater understanding of mechanisms underlying the transition from normal glycaemia to IGR and T2DM. This in turn could potentially inform screening and more targeted therapeutic interventions in the future.

## 6. Novelty Statement

- Using a biological proteomic ‘fingerprinting’ technique (SWATH MS) on plasma samples we set out to identify impaired glucose regulaton (IGR) individuals who were more likely to lose weight with a validated lifestyle change intervention.
- 20 people with IGR engaged in a six month lifestyle change intervention with samples taken pre- and post-intervention for proteomic evaluation.
- SWATH MS determined a panel of protein differences in people who were more likely to lose ≥3% in weight over the six month intervention period.
- Higher levels of insulin-like growth factor-II (IGF-II) were found to be predictive of greater success with weight reduction.

## Figures and Tables

**Table 1:** Anthropometric measurements of participants before and after the intervention.

	Male (n = 10) Mean ± Std. Error		Female (n = 10) Mean ± Std. Error		*p* Value
	Before	After	Before	After	
**Age (years)**	61.5 (4.83)		60.2 (2.73)		
**Weight (Kg)**	108.2 (9.18)	106.9 (9.26)	94.8 (5.16)	91.56 * (5.48)	0.02
**Height (cm)**	175.8 * (2.85)		161.9 (2.46)		0.002
**BMI**	35.0 (2.88)	34.6 (3.00)	36.2 (1.94)	35.06 * (2.11)	0.03
**Waist-hip**	1.00 (0.02)	1.00 (0.02)	0.90 (0.01)	0.90 (0.01)	

* *p* ≤ 0.05. BMI-body mass index.

**Table 2:** Clinical variables changes following intervention.

	Before Mean ± Std. Error	After Mean ± Std. Error	*p* Value
**HDL (mmol/L)**	1.37 (0.16)	1.31 (0.08)	0.685
**LDL (mmol/L)**	2.75 (0.26)	2.66 (0.27)	0.593
**Triglycerides (mmol/L)**	1.5 (0.19)	1.62 (0.16)	0.464
**Insulin (pmol/L)**	190.80 (58.12)	270.93 (131.58)	0.482

HDL-high-density lipoprotein, LDL-low-density lipoprotein.

**Table 3:** Change from baseline in plasma concentrations of growth factors.

	Before Mean ± Std. Error	After Mean ± Std. Error	*p* Value
**IGF-I (nmol/L)**	15.7 (1.31)	15.20 (1.29)	0.291
**IGF-II (nmol/L)**	70.4 (4.68)	66.9 (4.46)	0.166
**IGFBP3 (nmol/L)**	126.0 (8.11)	114.8 (7.59)	0.003 *
**IGF-i:IGFBP3 ratio**	0.126 (0.009)	0.133 (0.008)	0.033 *

* p ≤ 0.05. IGF-Insulin-like growth factor.

## Supplementary Materials

The following are available online at http://www.mdpi.com/2077-0383/8/2/141/s1, Table S1: List of the proteins analysed in the PCA discriminating future weight loss vs no significant weight loss included IGF-II and Vitamin D binding protein.
