# Supplementary material for: Data Independent Acquisition Mass Spectrometry Can Identify Circulating Proteins That Predict Future Weight Loss with a Diet and Exercise Programme
Source: J Clin Med. 2019 Jan 25;8(2):141. doi: 10.3390/jcm8020141 (PMC6406968; doi:10.3390/jcm8020141)
Supplement: Supplementary file 1 [file jcm-08-00141-s001.pdf]

Article

Data Independent Acquisition Mass Spectrometry Can Identify Circulating Proteins That Predict Future Weight Loss with a Diet and Exercise Programme

Nagaraj Malipatil [1,2], Helene A. Fachim [1,2,*], Kirk Siddals [1,2], Bethany Geary [1,3], Gwen Wark [4], Nick Porter [4], Simon Anderson [1], Rachelle Donn [1], Michelle Harvie [5,6,7], Anthony D. Whetton [1,3], Martin J. Gibson [1,2] and Adrian Heald [1,2,*]

1 The School of Medical Sciences and Manchester Academic Health Sciences Centre, University of Manchester, Manchester, M13 9PL, UK; nagaraj.malipatil@nhs.net (N.M.), kirk.siddals@manchester.ac.uk (K.S.), bethany.geary@manchester.ac.uk (B.G.), simon.anderson@manchester.ac.uk (S.A.), rachelle.donn@manchester.ac.uk (R.D.), tony.whetton@manchester.ac.uk (A.D.W.), martin.gibson@manchester.ac.uk (M.J.G.)

2 Department of Diabetes and Endocrinology, Salford Royal Hospital, Salford, M6 8HD, UK

3 Stoller Biomarker Discovery Centre, University of Manchester, Manchester, M13 9PL, UK

4 SAS Peptide Hormone Section, Part of Berkshire and Surrey Pathology Services, Royal Surrey County Hospital, Guildford GU2 7XX, UK; gwen.wark@nhs.net (G.W.), nick.porter@nhs.net (N.P.)

5 The Prevent Breast Cancer Research Unit, The Nightingale Centre, Manchester University NHS Foundation Trust, Manchester, M23 9LT, UK; michelle.harvie@manchester.ac.uk (M.H.)

6 Manchester Breast Centre, Manchester Cancer Research Centre, University of Manchester, 555 Wilmslow Rd, Manchester, M20 4GJ, UK

7 NIHR Manchester Biomedical Research Centre, University of Manchester, Manchester, M13 9WU, UK

* Correspondence: helene.fachim@manchester.ac.uk (H.A.F), adrian.heald@manchester.ac.uk (A.H.); Tel.: +44 161 206 5157 (A.H.)

Table S1. List of the proteins analysed in the PCA discriminating future weight loss vs no significant weight loss included IGF-II and Vitamin D binding protein.

Uniprot Entry Name	Protein Name
CISY_HUMAN	Citrate synthase, mitochondrial (EC 2.3.3.1) (Citrate (Si)-synthase)
FCN3_HUMAN	Ficolin-3 (Collagen/fibrinogen domain-containing lectin 3 p35) (Collagen/fibrinogen domain-containing protein 3) (Hakata antigen)
FA10_HUMAN	Coagulation factor X (EC 3.4.21.6) (Stuart factor) (Stuart-Prower factor) [Cleaved into: Factor X light chain; Factor X heavy chain; Activated factor Xa heavy chain]
CO3_HUMAN	Complement C3 (C3 and PZP-like alpha-2-macroglobulin domain-containing protein 1) [Cleaved into: Complement C3 beta chain; C3-beta-c (C3bc); Complement C3 alpha chain; C3a anaphylatoxin; Acylation stimulating protein (ASP) (C3adesArg); Complement C3b alpha' chain; Complement C3c alpha' chain fragment 1; Complement C3dg fragment; Complement C3g fragment; Complement C3d fragment; Complement C3f fragment; Complement C3c alpha' chain fragment 2]
IGF2_HUMAN	Insulin-like growth factor II (IGF-II) (Somatomedin-A) (T3M-11-derived growth factor) [Cleaved into: Insulin-like growth factor II; Insulin-like growth factor II Ala-25 Del; Preptin]
C1QC_HUMAN	Complement C1q subcomponent subunit C
CO9_HUMAN	Complement component C9 [Cleaved into: Complement component C9a; Complement component C9b]
RET4_HUMAN	Retinol-binding protein 4 (Plasma retinol-binding protein) (PRBP) (RBP) [Cleaved into: Plasma retinol-binding protein(1-182); Plasma retinol-binding protein(1-181); Plasma retinol-binding protein(1-179); Plasma retinol-binding protein(1-176)]
AMBP_HUMAN	Protein AMBP [Cleaved into: Alpha-1-microglobulin (Protein HC) (Alpha-1 microglycoprotein) (Complex-forming glycoprotein heterogeneous in charge); Inter-alpha-trypsin inhibitor light chain (ITI-LC) (Bikunin) (EDC1) (HI-30) (Uronic-acid-rich protein); Trypstatin]
FETUA_HUMAN	Alpha-2-HS-glycoprotein (Alpha-2-Z-globulin) (Ba-alpha-2-glycoprotein) (Fetuin-A) [Cleaved into: Alpha-2-HS-glycoprotein chain A; Alpha-2-HS-glycoprotein chain B]
HEMO_HUMAN	Hemopexin (Beta-1B-glycoprotein)
HRG_HUMAN	Histidine-rich glycoprotein (Histidine-proline-rich glycoprotein) (HPRG)
IC1_HUMAN	Plasma protease C1 inhibitor (C1 Inh) (C1Inh) (C1 esterase inhibitor) (C1-inhibiting factor) (Serpin G1)
CO4A_HUMAN	Complement C4-A (Acidic complement C4) (C3 and PZP-like alpha-2-macroglobulin domain-containing protein 2) [Cleaved into: Complement C4 beta chain; Complement C4-A alpha chain; C4a anaphylatoxin; C4b-A; C4d-A; Complement C4 gamma chain]
ZA2G_HUMAN	Zinc-alpha-2-glycoprotein (Zn-alpha-2-GP) (Zn-alpha-2-glycoprotein)
KAIN_HUMAN	Kallistatin (Kallikrein inhibitor) (Peptidase inhibitor 4) (PI-4) (Serpin A4)
HSP74_HUMAN	Heat shock 70 kDa protein 4 (HSP70RY) (Heat shock 70-related protein APG-2)
NAMPT_HUMAN	Nicotinamide phosphoribosyltransferase (NAmPRTase) (Nampt) (EC 2.4.2.12) (Pre-B-cell colony-enhancing factor 1) (Pre-B cell-enhancing factor) (Visfatin)

FAS_HUMAN	Fatty acid synthase (EC 2.3.1.85) [Includes: [Acyl-carrier-protein] S-acetyltransferase (EC 2.3.1.38); [Acyl-carrier-protein] S-malonyltransferase (EC 2.3.1.39); 3-oxoacyl-[acyl-carrier-protein] synthase (EC 2.3.1.41); 3-oxoacyl-[acyl-carrier-protein] reductase (EC 1.1.1.100); 3-hydroxyacyl-[acyl-carrier-protein] dehydratase (EC 4.2.1.59); Enoyl-[acyl-carrier-protein] reductase (EC 1.3.1.39); Oleoyl-[acyl-carrier-protein] hydrolase (EC 3.1.2.14)]
SYAC_HUMAN	Alanine--tRNA ligase, cytoplasmic (EC 6.1.1.7) (Alanyl-tRNA synthetase) (AlaRS) (Renal carcinoma antigen NY-REN-42)
TCPD_HUMAN	T-complex protein 1 subunit delta (TCP-1-delta) (CCT-delta) (Stimulator of TAR RNA-binding)
SYRC_HUMAN	Arginine--tRNA ligase, cytoplasmic (EC 6.1.1.19) (Arginyl-tRNA synthetase) (ArgRS)
AHNK_HUMAN	Neuroblast differentiation-associated protein AHNAK (Desmoyokin)
HABP2_HUMAN	Hyaluronan-binding protein 2 (EC 3.4.21.-) (Factor VII-activating protease) (Factor seven-activating protease) (FSAP) (Hepatocyte growth factor activator-like protein) (Plasma hyaluronan-binding protein) [Cleaved into: Hyaluronan-binding protein 2 50 kDa heavy chain; Hyaluronan-binding protein 2 50 kDa heavy chain alternate form; Hyaluronan-binding protein 2 27 kDa light chain; Hyaluronan-binding protein 2 27 kDa light chain alternate form]
NNRE_HUMAN	NAD(P)H-hydrate epimerase (EC 5.1.99.6) (Apolipoprotein A-I-binding protein) (AI-BP) (NAD(P)HX epimerase) (YjeF N-terminal domain-containing protein 1) (YjeF_N1)
PRG4_HUMAN	Proteoglycan 4 (Lubricin) (Megakaryocyte-stimulating factor) (Superficial zone proteoglycan) [Cleaved into: Proteoglycan 4 C-terminal part]
MELPH_HUMAN	Melanophilin (Exophilin-3) (Slp homolog lacking C2 domains a) (SlaC2-a) (Synaptotagmin-like protein 2a)
EPCR_HUMAN	Endothelial protein C receptor (Activated protein C receptor) (APC receptor) (Endothelial cell protein C receptor) (CD antigen CD201)
